# Ocular Ischemic Syndrome and Its Related Experimental Models

**DOI:** 10.3390/ijms23095249

**Published:** 2022-05-08

**Authors:** Deokho Lee, Yohei Tomita, Lizhu Yang, Kazuno Negishi, Toshihide Kurihara

**Affiliations:** 1 Laboratory of Photobiology, Keio University School of Medicine, Tokyo 160-8582, Japan; deokholee@keio.jp (D.L.); yohei.tomita@childrens.harvard.edu (Y.T.); yanglz@keio.jp (L.Y.); 2 Department of Ophthalmology, Keio University School of Medicine, Tokyo 160-8582, Japan; kazunonegishi@keio.jp; 3 Harvard Medical School, Boston Children’s Hospital, Boston, MA 02115, USA

**Keywords:** experimental model, ocular ischemia, ocular ischemic syndrome, carotid artery, ophthalmic artery

## Abstract

Ocular ischemic syndrome (OIS) is one of the severe ocular disorders occurring from stenosis or occlusion of the carotid arteries. As the ophthalmic artery is derived from the branch of the carotid artery, stenosis or occlusion of the carotid arteries could induce chronic ocular hypoperfusion, finally leading to the development of OIS. To date, the pathophysiology of OIS is still not clearly unraveled. To better explore the pathophysiology of OIS, several experimental models have been developed in rats and mice. Surgical occlusion or stenosis of common carotid arteries or internal carotid arteries was conducted bilaterally or unilaterally for model development. In this regard, final ischemic outcomes in the eye varied depending on the surgical procedure, even though similar findings on ocular hypoperfusion could be observed. In the current review, we provide an overview of the pathophysiology of OIS from various experimental models, as well as several clinical cases. Moreover, we cover the status of current therapies for OIS along with promising preclinical treatments with recent advances. Our review will enable more comprehensive therapeutic approaches to prevent the development and/or progression of OIS.

## 1. Ocular Ischemic Syndrome (OIS)

**Figure 1:**
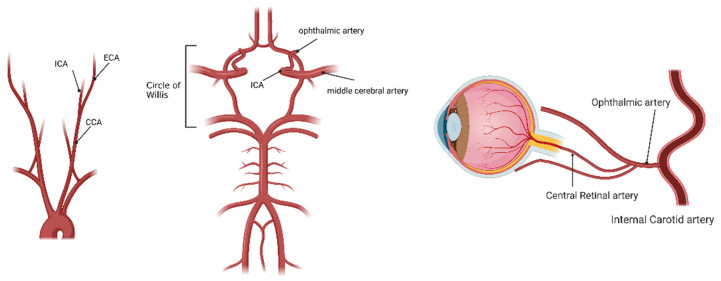
Ocular ischemic syndrome (OIS) development. OIS is caused by blocking the ophthalmic artery, the first branch of the internal carotid artery. CCA, the common carotid artery; ICA, the internal carotid artery; ECA, the external carotid artery. Image made with graphics from ©BioRender (biorender.com).

Ocular ischemic syndrome (OIS) is one of the ocular disorders caused by severe carotid artery occlusion or stenosis leading to hypoperfusion [1,2]. OIS occurs when there is a blockage of the ophthalmic artery, the branch of the internal carotid artery (ICA) (Figure 1). OIS is particularly common in patients with deficient collateral circulation between the left and right ICA or between the ICA and the external carotid artery (ECA). Patients with good collateral circulation who even have occlusion of the ICA may not develop OIS. However, patients with poor collateral circulation may develop OIS with less than 50% ICA stenosis [3]. Hedges reported diseases associated with ICA occlusion in 1963, and 20% of cases were caused by bilateral occlusions [4]. OIS is diagnosed in about 7.5 cases per million per year [1]. It is commonly caused in aging patients with diabetes, hypertension, and hyperlipidemia. Atherosclerosis, carotid artery dissection, trauma, and giant cell arteritis can sometimes cause OIS. 

Imaging the carotid artery is a crucial diagnostic test in OIS. As noninvasive tests, duplex carotid ultrasonography and Doppler ultrasonography are often used. Duplex carotid ultrasonography provides both flow velocity information and the imaging of the vessel. Compared with conventional digital subtraction angiography (DSA), Duplex ultrasound has higher sensitivity and specificity in detecting high-grade symptomatic carotid artery stenosis. Color Doppler imaging is a helpful adjunct to conventional duplex ultrasound [5]. Analyzing hemodynamic changes with Doppler images in the retrobulbar vessels can provide important information about the circulation [6]. 

**Figure 2:**
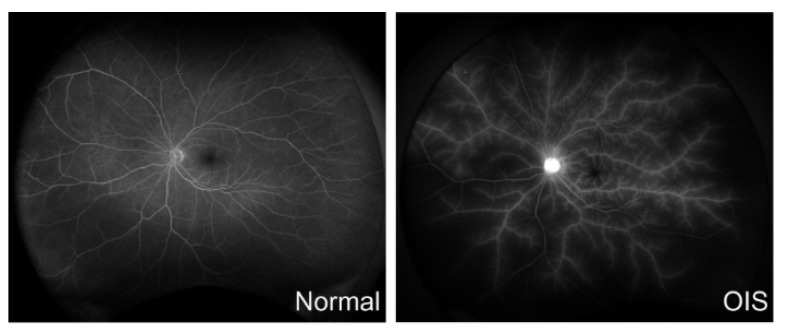
Fluorescein angiography (FA) in ocular ischemic syndrome (OIS). Delayed retinal circulation and hypoperfused areas are seen in OIS. There is also diffusion resembling frosted branch angiitis along all the retinal vessels. (**Left**): normal, 50 years old, 53 s; (**Right**): OIS, 79 years old, 48 s.

As an invasive test, carotid angiography is often used for the diagnosis. Carotid angiography is performed in advanced cases and before planning carotid surgery [1]. Angiography with magnetic resonance (MR) or computed tomography (CT) is also helpful to diagnose carotid artery stenosis [7]. Fundus fluorescein angiography is also a commonly used test for diagnosing OIS (Figure 2). Prolonged circulation time of the arm to choroid and the arm to retina is a frequent sign; approximately 60% of patients with OIS have irregular and prolonged filling times. This is the specific fluorescein angiographic sign of OIS [7,8]. Indocyanine green angiography can also help to confirm the diagnosis of OIS. This test can detect abnormalities in the choroidal circulation. Like fluorescein angiography, the prolonged circulation time of the arm to choroid can be seen in OIS patients using this test [7]. Electroretinography (ERG) can also assist in the diagnosis. Because the lack of both retinal and choroidal circulation in the OIS eyes causes the inner and outer retina to be ischemic, the amplitudes of both a- and b-waves are reduced [8].

The typical symptoms are visual loss and orbital pain [8,9,10,11,12,13,14]. Acute findings can be seen as ischemic transit attacks because of a transient decrease in central retinal artery blood flow. It is essential to review the patient’s history to avoid missing OIS because the duration of the attack is only a few minutes, after which vision improves. Chronic findings include capillary aneurysms, cotton-wool spots, cherry-red spots, and neovascularization at the optic disc. In addition, vitreous hemorrhage has been reported in 4% of patients [8]. Although 68% of patients develop neovascular glaucoma (NVG), some cases do not show high intraocular pressure (IOP) because of decreased humor production in the ciliary body [1,15]. The visual prognosis of OIS is poor, with 58% of corrected visual acuity below the finger count at 1 year [1]. The mortality rate within 5 years of onset is 40% [8,9,10,11,12,13,14]. Since cardiovascular disease is the leading cause of death, followed by stroke, patients with OIS should be referred to cardiologists and vascular surgeons, as well as neurologists. However, the efficacy of the current treatments is limited, and the mechanisms of OIS have not yet been fully understood.

## 2. Experimental Models

**Figure 3:**
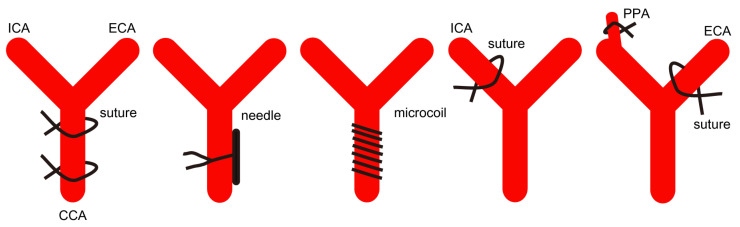
Experimental models of OIS suggested in the current review article. There are various methods of developing experimental models of OIS using unilateral or bilateral sutures, needles, and microcoils in mice and rats. Occlusion sites also vary depending on the studies, as discussed in Section 2. CCA, the common carotid artery; ICA, the internal carotid artery; ECA, the external carotid artery; PPA, the pterygopalatine artery.

Even though there are differences between humans and rodents, they share general physiological and anatomical aspects. In this regard, rodents, especially rats and mice, have been the leading model organisms used in experimental OIS models. Furthermore, as OIS commonly occurs when chronic and severe carotid artery obstruction exists [1,2], the use of in vitro models has limitations for understanding the pathophysiology of OIS. In this section, we cover pathological outcomes from experimental rodent models of OIS using occlusions of the cardiovascular system, which have similarities with the development of OIS in humans (Figure 3 and Table 1).

### 2.1. Bilateral Common Carotid Artery Occlusion/Stenosis (BCCAO/BCCAS)

Bilateral occlusions of common carotid arteries are generally performed in rats to develop chronic cerebral hypoperfusion-related neurodegenerative disorders and diseases [34]. Rats are a suitable rodent species as they have the complete circle of Willis (CoW) [35,36,37], which means that rats may not suddenly die after BCCAO. In contrast, the inadequate development of posterior communicating arteries in CoW causes dramatic cerebral ischemia in mice [38,39,40], finally leading to sudden death during/after BCCAO [29,41,42,43].

There have been various outcomes and aspects regarding ocular ischemia in BCCAO-operated rats. Leahy et al. demonstrated that artery diameter gradually increased in the retina of Long–Evans rats after BCCAO [16]. Vein diameter also increased in the retina of Long–Evans rats after BCCAO. Vein velocity dramatically decreased in 3 h after BCCAO, and its value gradually returned toward the value of vein velocity in sham-operated rats for 14 days. However, the value in BCCAO-operated rats was still significantly lower than that in sham-operated rats on day 14 after BCCAO. Total retinal blood flow trend was similar to the vein velocity trend in BCCAO-operated rats. Vascular oxygen content was measured in the retina of Long–Evans rats after BCCAO. Arterial oxygen content in BCCAO-operated rats was lower than that in sham-operated rats. Venous oxygen content was also lower than that in sham-operated rats. Arterio-venous oxygen content difference in BCCAO-operated rats was significantly higher than that in sham-operated rats. Lastly, oxygen delivery and oxygen metabolism in BCCAO-operated rats were significantly lower than that in sham-operated rats. Huang et al. positioned different sizes of needles (diameters of needles: 0.4, 0.6, and 0.9 mm) beside the common carotid arteries of Clean-grade Wister rats to induce effective carotid stenosis [17]. They found that needles with a diameter of 0.4 mm were the most powerful in developing severe stenosis of the carotid arteries [17]. In this system, they found that artery filling time significantly became longer after BCCAO, analyzed by fluorescein fundus angiography. Furthermore, blood flow, including the pupil, iris, and total eye, dramatically decreased in BCCAO-operated rats, analyzed by laser Doppler flowmetry. On the basis of these studies, the pathophysiology of ocular ischemia by BCCAO regarding oxygen delivery and the metabolism could be better understood.

Ocular functional and morphologic changes by BCCAO have been studied in various types of rats. Qin et al. demonstrated that amplitudes of scotopic a-wave and b-wave significantly decreased after BCCAO in Sprague–Dawley rats [18]. Furthermore, the latency of scotopic a-wave and b-wave was significantly delayed. Decreases in retinal thickness (including the total retinal layer from the inner membrane to the outer membrane, the inner nuclear layer, and the outer nuclear layer) were detected after BCCAO. Under transmission electron microscopy (TEM), disorder and damage in retinal ganglion cells (especially karyopyknosis, chromatic agglutination, and decreased or swelling organelles) were observed after BCCAO. Those retinal ganglion cells were surrounded by proliferating microglia, implying that microglia may affect retinal ganglion cell loss under the BCCAO-operated ocular ischemic condition [44,45]. In outer retinal cells, sizes of the nuclei became varied, the nuclear membranes were shrunken and invaginated, and densities of the chromatin became uneven. The outer segments of photoreceptor cells were loose, fractured, and dissolved. 

Sivilia et al. demonstrated that BCCAO caused pupillary light reflex (PLR) loss within 1 day after BCCAO in 62% of the surgery-operated Sprague–Dawley rats [19]. The loss of the PLR was permanent. Next, they found that the outer plexiform layer almost disappeared, and the inner plexiform and ganglion cell layers significantly decreased in BCCAO-operated rats after BCCAO, while the outer nuclear and inner nuclear layers were not significantly changed. This finding in Sivilia et al. [19] is slightly different from that in Qin et al. [18]. A significant reduction in the number of retinal ganglion cells was only found 75 days after BCCAO, not 8 or 30 days after BCCAO. Atrophy of the optic nerve, as analyzed by the optic nerve diameter, was observed 30 and 75 days after BCCAO, not 8 days after BCCAO. Lavinsky et al. divided BCCAO-operated Wistar rats into two groups (with or without the loss of the PLR by BCCAO) [20], as the PLR is associated with the severity of optic nerve damage [46]. They found that BCCAO-operated rats with loss of the PLR had more impairment in retinal thickness and ganglion cell density than BCCAO-operated rats without the loss of the PLR [20]. A similar study was performed by Davidson et al. using BCCAO-operated Sprague–Dawley rats [21]. This implies that the PLR could be a useful parameter for indicating a functional sign of ocular damage, including optic nerve damage. Furthermore, it suggests that this parameter may need to be considered for assessing therapeutic success in preclinical OIS studies.

Yamamoto et al. performed immunohistochemistry with various pro- and antiapoptotic factors and retinal cell markers in BCCAO-operated Wistar rats and examined their alterations in the ischemic retina [22]. Cleaved caspase-3-positive cells were clearly detected in the ganglion cell layer after BCCAO. Ubiquitin labeling intensity in the ganglion cell layer dramatically decreased. Cyclooxygenase-2 (COX-2) expression increased in the retina after BCCAO. The expression of heat-shock protein 70 (HSP70) transiently increased in 3 h after BCCAO and returned to the basal level within 24 h. When it comes to loss/damage of retinal cell types, parvalbumin-positive amacrine cells decreased after BCCAO. Choline acetyltransferase (ChAT)-positive cells in the ganglion cell layer were not changed for 6 months. Calbindin-positive horizontal cells dramatically decreased 1 week after BCCAO, and BRN3-positive cells in the ganglion cell layer significantly decreased 1 week after BCCAO. Furthermore, decreases in microtubule-associated protein 2 (one of the dendritic markers) and synaptophysin (one of the synaptic markers) were detected. Similar to this study, Chidlow et al. aimed to unravel molecular changes (especially focusing on HSPs) in the ischemic retina after BCCAO [23]. They found significant increases in *Hsp27* and *Hsp70* mRNA expressions in the ischemic retina of Sprague–Dawley rats on days 2 and 7 after BCCAO. While HSP70 protein expression did not increase in the retina and optic nerve on day 7 after BCCAO, HSP27 protein expression significantly increased in the retina and optic nerve on the same day. Its expression was highly detected in the ganglion cell and inner plexiform layers. Another study from Chidlow et al. showed a series of optic nerve degeneration using the same model [24]. Gradual increases in HSP27 and αB-crystallin expression and gradual decreases in NFL and β3-tubulin protein expression were found in the proximal optic nerve after BCCAO. Furthermore, they found that deposition of extracellular matrix components (collagen I, collagen VI, and laminin) occurred within the optic nerve head and proximal optic nerve. Abnormal labeling of extracellular matrix components was evident on 1 week, pronounced on 2 weeks, and extensive on 3 weeks after BCCAO. On the basis of these studies, multiple mechanisms of BCCAO-induced ischemic damage to retinal cells and the optic nerve could be further unraveled at the molecular level. 

Wang et al. examined the feasibility of induction of OIS in spontaneously hypertensive rats [25]. Using hypertensive and normotensive Wistar–Kyoto rats, they found high dysfunction rates in the PLR in BCCAO-operated spontaneously hypertensive rats. Furthermore, those rats had extensive avascular areas of blood vessels and dramatic decreases in retinal thickness. This implies that systemic hypertension may enhance BCCAO-induced ocular ischemic injuries. This could be connected with the clinical record that hypertension was found in 50% to 73% of OIS patients [14,47]. Holman et al. screened therapeutic or detrimental effects of hyperglycemia in BCCAO-operated Sprague–Dawley rats [26]. Hyperglycemia was induced by the intraperitoneal injection of streptozotocin 3 days before BCCAO. Then, the retinal changes were examined on day 7 after BCCAO, representing a short-term investigation. BCCAO-operated hyperglycemic rats showed a reduction in retinal protection (such as preservation of retinal thickness and survival of BRN3-, Islet-1-, PGP 9.5-, and calbindin-positive retinal cells) and pathological gliosis (such as activation of astrocytes, Müller cells, and microglia) compared with those in BCCAO-operated normoglycemic rats. Even though more studies are needed for long-term investigation, short-term hyperglycemia could have a chance of preventing retinal cell death from BCCAO-induced retinal hypoperfusion injuries. 

Even though techniques of BCCAO and BCCAS are relatively difficult to be applied to mice as they die during/after the operation, Crespo-Garcia et al. used the coil implantation method on male C57BL/6J mice at the age of 10 weeks and developed a mouse model of BCCAO/BCCAS-induced OIS [27]. BCCAO/BCCAS induced significant retinal vein dilatation after BCCAO/BCCAS. Inflammatory dynamics was detected in the ischemic retina, using MacGreen mice to monitor mononuclear phagocytes and a quantitative PCR method (*Ccl2*, *Cd68*, and *Il-1β*). Mobilization and accumulation of mononuclear phagocytes were observed in surrounding veins. Furthermore, they found that amplitudes in scotopic a-wave, b-wave, and oscillatory potentials gradually decreased in the ischemic retina after BCCAO/BCCAS. Similarly to BCCAO in rats, retinal pathological gliosis and retinal thinning occurred in mice. Synaptic degeneration (evaluated by vesicular glutamate transporter 1, C-terminal-binding protein 2, protein kinase C-α, and calbindin-D28k) was detected in the retina from 1 week to 6 weeks after BCCAO/BCCAS. Taken together, outcomes and aspects regarding ocular ischemia in murine models of OIS by BCCAO/BCCAS are stacked.

### 2.2. Unilateral Common Carotid Artery Occlusion (UCCAO)

As mice are known to be more susceptible to ischemic injuries than rats [40,48,49], researchers applied the method of unilateral occlusion of the common carotid artery to mice. Even though only one eye in each mouse is available for the ischemic eye by UCCAO, researchers have become better able to understand the pathological mechanisms of OIS using mice in terms of convenient handling [50,51]. 

Lee et al. found that retinal hypoxia-inducible factor-2α (HIF-2α) protein was stabilized in C57BL/6 mice after UCCAO [28]. The mRNA expression of *Epo* significantly increased after UCCAO. Furthermore, they demonstrated that astrocytic cystine/glutamate antiporter may be an important regulator of EPO expression in the ischemic retina (especially in the retinal ganglion cell layer). Even though the characterization of the model itself was not extensively performed, the status of hypoxia-regulated EPO gene expression was better understood in UCCAO-operated mice. 

To solve this issue, Lee and Kang et al. executed the model characterization from the early stage to the chronic stage after UCCAO in C57BL/6 mice [29]. UCCAO caused abnormal retinal blood perfusion analyzed by FITC–dextran labeling after UCCAO. Eyelid drooping was detected after UCCAO, and this finding was chronically observed. The stabilization in retinal HIF-1α protein was detected after UCCAO, and GFAP-positive active gliosis was seen at the early stage (1–3 days after UCCAO). While retinal thinning was not dramatically observed until 10 weeks after UCCAO (analyzed with H&E-stained retinal sections), reductions in the mRNA and protein expressions of THY1 (one of the inner retinal cell markers) were detected after UCCAO. The death rate during/after UCCAO was 0% during the study. 

However, as retinal functional changes have not been clearly studied in previous studies above, we (Lee and Jeong et al.) used the same model (UCCAO-operated C57BL/6 mice) to examine whether UCCAO could induce retinal functional impairments [30]. Using ERG, we demonstrated that the b-wave amplitude dramatically decreased while that of the a-wave was slightly changed 7 days after UCCAO. Furthermore, using optical coherence tomography (OCT), we demonstrated that retinal thickness was not significantly altered by UCCAO even though there were fluctuations in retinal thickness for 14 days. Other cellular changes (such as chronic retinal gliosis and transient retinal cell death) were observed for 14 days. On the basis of these studies, pathological outcomes using a mouse model of UCCAO were better established. 

To expand the understanding of the series of ocular degeneration by UCCAO in mice, we (Lee et al.) aimed to screen unfound parameters in the same model [31]. In UCCAO-operated mice, the function of the PLR or IOP was not affected. Acute reversible cataract by anesthesia in UCCAO-operated mice was detected for a shorter time than that in sham-operated mice. Cerebral blood flow (analyzed by laser speckle flowmetry) and retinal blood flow (analyzed by FITC–dextran labeling) were reduced right after UCCAO. The mRNA expressions of hypoxia-response genes (*Vegf* and *Bnip3*) significantly increased in the retina, and only *Bnip3* mRNA expression increased in the choroid/retinal pigment epithelium (RPE). Along with a decrease in scotopic b-wave, photopic b-wave was also damaged by UCCAO at 2 and 4 weeks. The amplitudes of visual evoked potential significantly decreased 2 and 4 weeks after UCCAO. NeuN-positive cell loss was detected in the ganglion cell layer 2 weeks after UCCAO. However, there were no dramatic decreases in thickness in the choroid/RPE for 4 weeks. When it comes to retinal inflammation, increases in isolectin-IB4-positive inflammatory cells and *Ccl2* mRNA expression were observed. Taken together, pathological outcomes of ocular ischemia in a mouse model of OIS by UCCAO are well established.

### 2.3. Occlusion(s) of the Other Branches of the Carotid Artery

The common carotid artery is one of the most vital blood vessels in organisms in that it directly determines life and death. Other than gross occlusion of the common carotid artery, more delicate work from the anatomical point of view aimed to develop murine models of OIS. Ling et al. used bilateral ICA occlusion (BICAO) on C57BL/6 mice [32]. Even though there was no specifically different outcome in BICAO-operated mice compared with that in other BCCAO/BCCAS-operated rats or mice introduced above, they used various methods including MR angiography, H&E staining, fluorescein angiography, and OCT to investigate pathophysiologic mechanisms of OIS in this model. Ogishima et al. occluded the pterygopalatine artery (PPA) and ECA to induce ischemic damage in the retina of ddY mice [33]. As the PPA supplies blood/oxygen to the eye and the ECA supports the vascular network between the PPA and the ophthalmic artery [40,52,53], a new model of OIS could be developed by occlusion of the PPA and ECA. Laser speckle imaging showed that ocular blood flow decreased 5 min after the occlusion surgery, and its flow in the occlusion-operated mice was still detected lower than that in sham-operated mice. Functional (analyzed by ERG) and histologic (analyzed by H&E staining) damage in the inner retina were similar with general BCCAO/BCCAS-operated rats or mice. According to FITC–dextran labeling analysis, they found that occlusion of only PPA or ECA did not efficiently block the retinal blood perfusion, while occlusion of both arteries achieved complete obstruction. Taken together, several trials have led to the development of new murine models of OIS using a more sophisticated occlusion method.

## 3. Therapeutics

### 3.1. Preclinical Studies

There have not been direct studies to develop promising therapeutics for OIS, as OIS has a broad spectrum of ocular diseases such as ocular neovascularization, rubeosis iridis, iris necrosis, and cataracts. Therefore, we cover this section after narrowing down the drug pool tested in various murine models of carotid artery occlusion-induced ocular ischemia, discussed in Section 2. 

Protective effects of recombinant human EPO (rhEPO) were tested by Zhou et al. in BCCAO-operated Sprague–Dawley rats [54]. Intranasal injection of rhEPO improved visual impairments. The amplitudes of visual evoked potential, retinal thickness, and retinal ganglion cells were preserved against BCCAO-induced ischemic injuries. Even though the mode of action of rhEPO was not experimentally described in the study, EPO (a well-known strong neuroprotective molecule in the central nervous system) may directly exert neuroprotection to the ischemic retina and/or brain.

Du et al. applied osthole for managing the hypoperfused retina in BCCAO-operated Brown Norway rats [55]. Osthole is a naturally occurring coumarin derivative used as traditional oriental medicine [56], and its therapeutic effects have been documented in various ischemic conditions [57,58,59]. Significant improvements in retinal thickness were noticed in osthole-administered rats, detected by H&E staining [55]. The number of TUNEL-positive cells was reduced by osthole administration. According to the immunohistochemistry analysis, they demonstrated that levels of Akt and NF-κB were lower while the ratio of BCL-2/BAX was higher in osthole-administered rats. These results imply that osthole may protect the retina against ocular ischemic injures by BCCAO via anti-inflammatory and antiapoptotic pathways. 

Sivilia et al. used neurotrophin nerve growth factor (NGF) via a single intravitreal injection against retinal degeneration by BCCAO in Sprague–Dawley rats [19]. According to the notion that NGF can improve the survival of neurons, neurite outgrowth, and control axonal receptivity to myelination [60,61,62,63], NGF administration controlled the demyelination/remyelination status in the optic nerve and protected against optic nerve atrophy and retinal ganglion cell degeneration by BCCAO in rats. 

A short report from Szabadfi et al. demonstrated that urocortin 2, a corticotropin-releasing factor (CRF) paralog activating CRF_2_ receptors, had protective effects against retinal degeneration (preservation of retinal thickness and protection of retinal ganglion cells) by BCCAO in Wistar rats without the mode of action of urocortin 2, requiring further investigation [64]. Similarly, Werling et al. only screened therapeutic effects of various neuropeptide pituitary adenylate cyclase activating polypeptide (PACAP) fragments in BCCAO-operated Wistar rats, and they found that PACAP 1-38 had the greatest efficacy against the injuries [65]. 

Previously, we (Lee and Tomita et al.) also screened drug candidates (fenofibrate and pemafibrate) in UCCAO-operated C57BL/6 mice for developing therapeutics in OIS [66,67]. These drugs are well-known peroxisome proliferator-activated receptor alpha (PPARα) agonists, and they have been increasingly nominated as promising drugs for various ischemic retinopathies with diabetes or hyperlipidemia [68,69]. Consecutive oral administrations of pemafibrate (before and after UCCAO) prevented reductions in the amplitudes of a-wave, b-wave, and oscillatory potentials 10 days after UCCAO [67]. Oral administrations of pemafibrate reduced pathological gliosis detected by GFAP on days 2 and 5 after UCCAO. The expressions in hepatic PPARα target genes (*Acox1*, *Fgf21*, *Fabp4*, *Vldlr*, and *Ucp3*) rather than those in ocular PPARα target genes were upregulated by oral administrations of pemafibrate. Fibroblast growth factor 21 (one of the strong neuroprotective molecules in the brain and eye [70,71,72,73,74]) was produced by oral administrations of pemafibrate and plausibly circulated to exert neuroprotection in the ischemic retina. Furthermore, oral administrations of pemafibrate reduced serum levels of triglyceride and upregulated serum levels of total cholesterol, which may have indirect effects on the protection. Similar findings were also detected in fenofibrate-administered UCCAO-operated mice [66]. As these two drugs are already available in the clinic, a future direction in OIS is also positively anticipated. Taken together, even though drug screening on ocular protection has been performed in various experimental models of carotid artery occlusion-induced ocular ischemia, further investigations are needed.

### 3.2. Current Clinical Treatment 

Treatment for OIS is usually limited to treating complications. The treatment can be divided into two aspects: ocular treatment and systemic treatment. 

#### 3.2.1. Ocular Treatment

Pan-retinal photocoagulation could reduce the demand for oxygen consumption in the ischemic retina to avoid NVG formation [75]. It could lead to regression of iris vascularization (NVI) in 36% of cases and control of IOP in patients with open-angle glaucoma [13]. However, there are many cases in which treatment is ineffective because VEGF is produced in the choroid and anterior segment, as well as the retina. Intravitreal anti-VEGF may be helpful to control the NV and macular edema associated with the disease.

Elevated IOP in OIS often resists medical treatment; drugs that reduce aqueous secretion, such as β blockers and α agonists, can be used. In some cases, trabeculectomy (TLE), a shunt procedure, may be necessary for patients with angle occlusion due to NV and the inability to control IOP. It has been reported that anti-VEGF drugs before TLE can suppress postoperative anterior chamber hemorrhage and improve visual function in the early postoperative period [76].

To treat inflammation in the anterior chamber, topical steroids can be used. In addition, eye drops of mydriatics are used to reduce inflammation in the anterior segment. However, these are only symptomatic treatments. Proinflammatory drugs such as prostaglandin and pilocarpine should be avoided [7].

#### 3.2.2. Systemic Treatment

For radical treatment, neurosurgical improvement in ICA blood flow is necessary, and carotid endarterectomy (CEA) and carotid artery stenting (CAS) are indicated. CEA is performed to remove the plaque from inside the artery and is known to reduce the risk of stroke in symptomatic patients [77]. CEA has been proven to increase blood flow in the ocular arteries and prevent ischemic changes [5]. Another group found that CEA improved ophthalmic blood flow with post-CEA values comparable to normative values in healthy patients [78]. In addition, they showed a correlation between visual symptoms and flow velocities before the CEA procedure [78]. A clinical trial showed the superiority of the combination of aspirin and CEA treatment in preventing stroke compared to aspirin only in patients with carotid artery stenosis [13].

**Figure 4:**
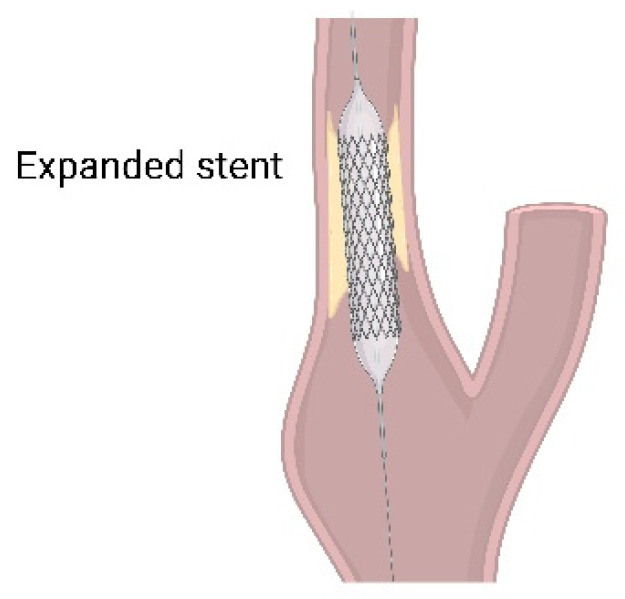
Carotid artery staining (CAS). Expanding stent is inserted into the carotid artery to increase blood flow blocked by plaque. Image made with graphics from ©BioRender (biorender.com).

On the other hand, CAS is a procedure in which an expanding stent is inserted into the carotid artery to increase blood flow to the area blocked by plaque (Figure 4). CAS is performed on patients for whom CEA may cause complications, such as those who have had previous radiation, neck surgery, recurrent stenosis, tracheostomy, or complicated surgery with stenosis above the second cervical (C2) vertebral level. High-risk patients such as those with unstable angina, congestive heart failure, and recent myocardial infarction are also indicated for stent [79]. It has been reported that there is no difference in the outcomes of CAS and CEA in terms of perioperative stroke, myocardial infarction, death, and 4 year incidence of ipsilateral stroke after the perioperative period [8].

Extracranial–intracranial (EC-IC) arterial bypass surgery involves the creation of an anastomosis between the extracranial branch of the superficial temporal artery and the intracranial branch of the tunica media artery. It is indicated when the ICA or CCA is completely occluded or when it is difficult to assess at the C2 level or higher because of stenosis. It can, thus, prevent cerebral ischemia [79]. However, even with surgical treatment, the prognosis for visual function is poor [14].

These procedures above could restore normal blood flow in the eye, and they are thought to stabilize visual acuity if performed before NVI formation. However, neurologists and internists should thoroughly evaluate patients with OIS because of numerous comorbidities in these patients. Any associated predisposing comorbidities should be treated, and patients are recommended to adopt an appropriate diet, stop smoking, and undertake physical activities. Furthermore, a group showed a case of retinal embolization ischemia following ICA stenting [80]. Thus, ophthalmologists need to monitor any retinal ischemic changes using fluorescein angiography and OCT, and patients should be informed about the risk of permanent vision loss even after receiving treatments.

## 4. Future Directions and Conclusions

In this review article, we covered basic knowledge of human OIS regarding epidemiology, symptoms, diagnosis, and treatment, and we summarized the pathophysiologic outcomes from experimental models of OIS through various carotid artery occlusion methods in rats and mice. Even though we combined pathologic outcomes from experimental models of OIS in this study, insights into therapeutic approaches and pathological mechanisms occurring in the eye under the condition of OIS are still limited. Therefore, we hope that our current summary contributes to a comprehensive understanding of pathophysiologic mechanisms of OIS and urges more preclinical and clinical research on unraveling pathophysiologic mechanisms and finding promising therapeutics for OIS in the near future.

## Figures and Tables

**Table 1:** Summary of experimental models of OIS.

Publication	Strain	Method	Main Outcome
Leahy et al., 2020 [16]	Long–Evans rats	BCCAO	Abnormal changes in artery/vein diameter, vein velocity, total retinal blood flow, and oxygen delivery and metabolism
Huang et al., 2014 [17]	Clean-grade Wister rats	BCCAO	Longer artery filling time; decreases in ocular blood flow (the pupil, iris, and total eye)
Qin et al., 2019 [18]	Sprague–Dawley rats	BCCAO	Decreases in amplitudes of scotopic a-wave and b-wave; decreases in retinal thickness; disorder and damage in retinal ganglion cells (especially karyopyknosis, chromatic agglutination, and decreased or swelling organelles); disorder and damage in photoreceptor cells
Sivilia et al., 2009 [19]	Sprague–Dawley rats	BCCAO	PLR loss; decreases in the outer plexiform layer and the inner plexiform and ganglion cell layers without affecting the outer nuclear and inner nuclear layers
Lavinsky et al., 2006 [20]	Wistar rats	BCCAO	PLR loss; more impairment in retinal thickness and ganglion cell density in BCCAO-operated rats with PLR loss
Davidson et al., 2000 [21]	Sprague–Dawley rats	BCCAO	PLR loss; general retinal damage and visual loss
Yamamoto et al., 2006 [22]	Wistar rats	BCCAO	Molecular alterations in various pro- and antiapoptotic factors and retinal cell markers such as cleaved caspase-3, ubiquitin, COX-2, HSP70, calbindin, BRN3, microtubule-associated protein 2, and synaptophysin
Chidlow et al., 2014 [23]	Sprague–Dawley rats	BCCAO	Increases in HSP27 protein expression in the retina and optic nerve (especially, in the ganglion cell and inner plexiform layers)
Chidlow et al., 2010 [24]	Sprague–Dawley rats	BCCAO	Gradual increases in HSP27 and αB-crystallin expressions and gradual decreases in NFL and β3-tubulin protein expressions found in the proximal optic nerve; deposition of extracellular matrix components (collagen I, collagen VI, and laminin)
Wang et al., 2016 [25]	Hypertensive and normotensive Wistar–Kyoto rats	BCCAO	Higher dysfunction rates in the PLR under hypertension; extensive avascular areas of blood vessels; dramatic decreases in retinal thickness
Holman et al., 2010 [26]	Sprague–Dawley rats with streptozotocin	BCCAO	Retinal protection (such as preservation of retinal thickness and survival of BRN3-, Islet-1-, PGP 9.5-, and calbindin-positive retinal cells); pathological gliosis (such as activation of astrocytes, Müller cells, and microglia) reduction by short-term hyperglycemia
Crespo-Garcia et al., 2018 [27]	C57BL/6J mice	BCCAO/BCCAS	Retinal vein dilatation; mobilization and accumulation of mononuclear phagocytes in surrounding veins; decreases in amplitudes in scotopic a-wave, b-wave, and oscillatory potentials; synaptic degeneration (vesicular glutamate transporter 1, C-terminal-binding protein 2, protein kinase C-α, and calbindin-D28k)
Lee et al., 2019 [28]	C57BL/6 mice	UCCAO	Retinal HIF-2α stabilization; increases in *Epo* mRNA expression; decreases in total retinal thickness
Lee and Kang et al., 2020 [29]	C57BL/6 mice	UCCAO	Abnormal retinal blood perfusion; eyelid drooping; retinal HIF-1α stabilization; acute GFAP-positive gliosis; chronic retinal thinning
Lee and Jeong et al., 2021 [30]	C57BL/6 mice	UCCAO	Decreases in amplitudes of scotopic b-wave; chronic retinal gliosis; transient retinal cell death
Lee et al., 2021 [31]	C57BL/6 mice	UCCAO	No change in PLR or IOP; acute reversible cataract development; visual evoked potential reduction; NeuN-positive cell loss; chronic retinal inflammation
Ling et al., 2017 [32]	C57BL/6 mice	BICAO	Abnormal ocular blood flow; alterations in retinal thickness
Ogishima et al., 2011 [33]	ddY mice	Occlusion of the PPA and ECA	Decreases in ocular blood flow; functional and histologic damage in the inner retina

## Data Availability

The data presented in this study are available on request from the corresponding author.
